# “Meet the IUPAB councilor”—Thomas Gutsmann

**DOI:** 10.1007/s12551-024-01226-1

**Published:** 2024-10-21

**Authors:** Thomas Gutsmann

**Affiliations:** Research Center Borstel, Leibniz Lung Center, Division of Biophysics, Center for Systems Structural Biology (CSSB), Hamburg, Parkallee 10, D-23845 Borstel, Germany

## Abstract

As one of the twelve newly elected councillors, it is my pleasure to provide a brief biographical sketch for the readers of Biophys. Rev. and the members of the Biophysical Societies. I have been actively involved in the German Biophysical Society (DGfB) since 2008, initially as the speaker for the “Membrane Biophysics” section and, since 2015, as the secretary. Within the IUPAB council I follow Prof. Hans-Joachim Galla, former Secretary and President of the German Biophysical Society, who served as a councillor for two terms from 2018 to 2024. Thus, a direct continuation of the German contribution to the IUPAB is guaranteed. My journey in biophysics began during my studies of physics at the University of Kiel, where I specialized in physiology and biophysics. After earning my doctorate in the lab of Ulrich Seydel at the Research Center Borstel, I spent two years at the University of California, Santa Barbara, working in Paul Hansma’s lab on the development and application of atomic force microscopy. During my time at UCSB, I also collaborated with Jacob Israelachvili’s lab on membrane properties. Since 2008, I have been leading the Biophysics Research Group at the Research Center Borstel, Leibniz Lung Center. In 2010, I was appointed as a professor at the University of Lübeck. Additionally, since 2023, I have been serving as an associate member at the Centre for Structural Systems Biology (CSSB) in Hamburg.


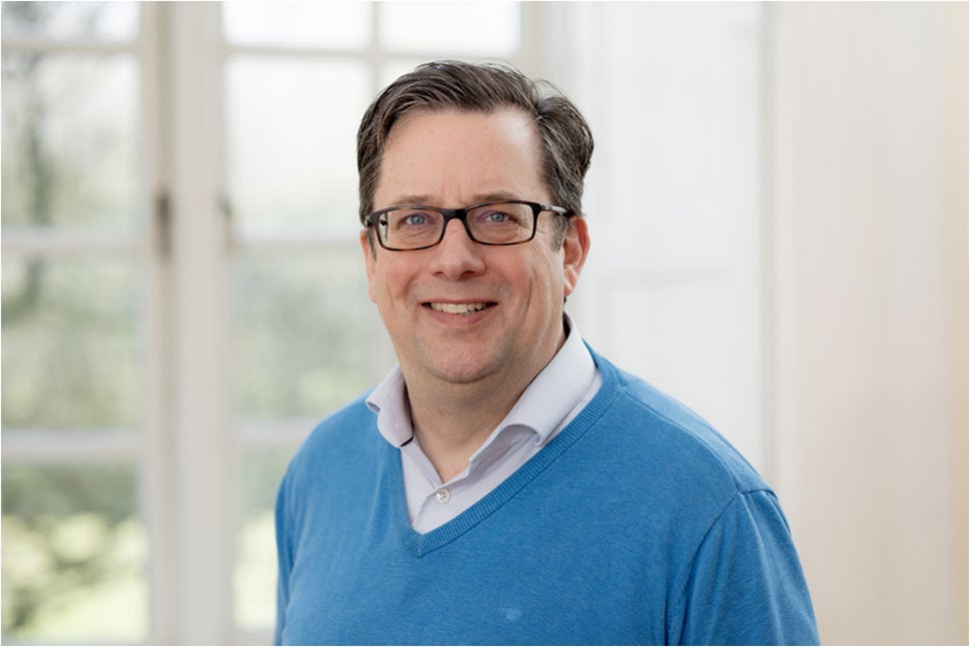


## Contributions to biophysics

My research focuses on the lipid membranes of pathogens and host cells, which play a central role in many infectious diseases. The biophysical properties of these membranes and the interactions between individual molecules and membranes are at the heart of the work in my lab at the Research Center Borstel, Leibniz Lung Center. We are particularly interested in questions regarding Gram-negative bacteria, mycobacteria, and nontuberculous mycobacteria (NTMs). Based on biomedical questions often developed in collaboration with national and international partners, we use various membrane models in combination with different biophysical methods (e.g., atomic force microscopy, electrophysiology, calorimetry, X-ray scattering, fluorescence spectroscopy, and microscopy) to decipher the mechanisms of action of membrane-active substances. In recent years, aerosols have also become a focus, where lipid membranes play a crucial role as well.

Our research has six major foci:

1. **Membrane structure**: The foundation of all investigations is the characterization of the lipid membrane. The electrical and mechanical properties of the membranes, as well as their lateral and transverse organization, are essential. We study these properties in both natural membranes and reconstituted systems. To achieve this, we have expertise in purifying lipids from natural membranes and the targeted reconstitution of membranes. To mimic bacterial and immune cell membranes, we have established new reconstitution systems, including microfluidic chip-based systems (Gutsmann et al. 2015). The bilayers consist of phospholipids, lipid II (Wiedemann et al. 2006), and lysyl-PG to mimic the membrane of Gram-positive bacteria and phospholipids and cholesterol to mimic the cytoplasmic membrane of eukaryotic cells. Additionally, we have reconstructed the outer membrane of Gram-negative bacteria as an asymmetric planar lipid bilayer and as asymmetric liposomes (Paulowski et al. 2020), with one side consisting of lipopolysaccharides (LPS) and the other of phospholipids. We succeeded in mimicking the mycobacterial wax layer with lipid matrices containing the glycolipid trehalose dimycolate (TDM).
2. **Pore-forming antimicrobial substances**: The antimicrobial peptides (AMPs) we have investigated in recent years differ in their structure and activity. Based on our hypothesis that lipid- and peptide-specific properties are responsible for the sensitivity or resistance of certain bacterial strains, we have characterized the interactions between the AMPs and reconstituted lipid membranes (Gutsmann et al. 1999; Gutsmann et al. 2000). For our investigations, we used different AMPs in their natural form as well as synthetic derivatives. We found an almost perfect correlation between the biological activity of the investigated AMPs and their interaction with pure lipid matrices (Gutsmann and Seydel 2010). Thus, in a first step, the permeabilization of the lipid membrane is an essential prerequisite for killing bacteria. Based on our findings, we synthesized peptides with improved activities. The endotoxin-neutralizing activity is achieved not only through the direct interaction of the AMPs with the endotoxin but also through the modification of the receptor complexes embedded in the host cell membrane (Paulowski et al. 2020; Schromm et al. 2021).
3. **Microbial toxins**: When microbes are ingested by professional phagocytes, different interactions can occur in their phagosomes. These effects can lead to the killing of bacteria or the survival of some intracellular microbes. There is evidence that phagocytes use pore-forming peptides of innate immunity, known as host defense peptides (HDP) or AMPs, to kill microbes. On the other hand, microbes use pore-forming proteins and peptides to prevent the acidification of the phagosome. We investigate the mechanisms of action of the mycobacterial ESAT6/CFP10 and other WXG proteins. Additionally, we have several collaborations for characterizing, e.g., candidalysin (Moyes et al. 2016) , PLAs, and VapA (Nehls et al. 2024). Understanding the species specificity plays a crucial role in understanding why AMPs and toxins target different membrane structures despite having very similar mechanisms of action.
4. **Microbial adhesion**: Bacteria and other microorganisms adhere to surfaces through various mechanisms. These surfaces can be host cells or technical surfaces such as implants. Due to our expertise in conducting binding measurements, we study the adhesion of malaria-infected erythrocytes to epithelial cells (Bachmann et al. 2022).
5. **Infectious aerosols**: We have significantly expanded our expertise in the field of aerosols in recent years (Pfrommer et al. 2020). Two projects are currently in focus:
  **DUSTRISK**: In this project, a large consortium of researchers from Germany and physicians in Cape Verde investigates the infectivity of desert dust particles. Our contribution here is the characterization of the aerosols and the adhesion of microbes to dust particles.
  **Aerofix**: In this project, we produce model aerosols, characterize them, and intend to develop a graphene-based sensor system for the rapid diagnostics of various airborne pathogens.
6. **Therapeutic aerosols**: To further advance the therapeutic application of AMPs, we have begun characterizing the interaction of peptides with lipid monolayers as a model of the surfactant monolayer in the lung. This requires the setup of a film balance with a nebulization system through which known drugs can be applied that are administered via the lung in therapeutic practice. Furthermore, we have developed a new device to characterize the mechanical stability of drug pellets administered as aerosols.
